# Profiling proteomic responses in small intestinal neuroendocrine tumor GOT1 after [^177^Lu]Lu-DOTATATE therapy

**DOI:** 10.1186/s12885-026-16975-3

**Published:** 2026-09-16

**Authors:** Johan K.E. Spetz, Mikael E. Montelius, Evelin Berger, Julia A.M. Johansson, Nishte Rassol, Maria Ljungberg, Eva B. Forssell-Aronsson

**Affiliations:** 1 Department of Medical Radiation Sciences, Institute of Clinical Sciences, Sahlgrenska Academy, University of Gothenburg, Gula Stråket 2B, 3rd floor, Sahlgrenska University Hospital, Gothenburg, SE-413 45 Sweden; 2 Sahlgrenska Center for Cancer Research, Sahlgrenska Academy, University of Gothenburg, Gothenburg, Sweden; 3 Department of Medical Physics and Biomedical Engineering, Sahlgrenska University Hospital, Region Västra Götaland, Gothenburg, Sweden; 4 SciLifeLab, Proteomics Core Facility, Sahlgrenska Academy, University of Gothenburg, Gothenburg, Sweden

**Keywords:** Radiation biology, Ionizing radiation, Mass spectrometry, GEPNET, PRRT, Lutathera

## Abstract

**Background:**

Peptide receptor radionuclide therapy with [^177^Lu]Lu-DOTATATE is an established treatment for somatostatin receptor–expressing neuroendocrine tumors. Although strong anti-tumor effects have been demonstrated in experimental models, curative responses in patients remain limited. Improved understanding of the molecular responses induced by [^177^Lu]Lu-DOTATATE may help identify strategies for treatment optimization. This study aimed to characterize proteomic alterations in GOT1 small intestinal neuroendocrine tumor xenografts following [^177^Lu]Lu-DOTATATE therapy.

**Methods:**

GOT1 tumor–bearing BALB/c nude mice received a non-curative intravenous administration of 15 MBq [^177^Lu]Lu-DOTATATE or saline control. Tumors were collected 1 or 13 days after treatment to represent early response and regrowth phases. Tumor volumes were monitored using magnetic resonance imaging. Proteomic profiling was performed using liquid chromatography tandem mass spectrometry with tandem mass tag labeling. Differential protein expression was analyzed using Welch’s t-test with significance defined as fold change ≥ 1.5 and *p* < 0.01. Functional enrichment and pathway analyses were conducted using Gene Ontology annotation and Ingenuity Pathway Analysis.

**Results:**

Treatment induced a transient reduction in tumor volume followed by regrowth. In total, 3861 proteins were quantified, of which 155 showed significantly altered expression after treatment. Affected proteins were associated with cytoskeletal organization, oxidative stress response, protein metabolism, and systemic regulation. Pathway analyses identified inhibition of several signaling pathways linked to cell migration and invasiveness, including RhoA, Rac, integrin, and CXCR4 signaling. Upstream regulator analysis suggested activation of RABL6 and inhibition of p53 signaling during tumor regrowth. Proteins related to endoplasmic reticulum stress and ubiquitin-proteasome activity were also altered. Selected findings were validated by ELISA.

**Conclusions:**

[^177^Lu]Lu-DOTATATE therapy induced extensive proteomic changes in GOT1 tumors, including suppression of pathways associated with invasiveness and modulation of p53- and stress-related signaling. These findings suggest potential therapeutic benefits of combining [^177^Lu]Lu-DOTATATE with agents targeting p53 regulation or endoplasmic reticulum stress pathways to improve treatment efficacy in neuroendocrine tumors.

## Background

Radiation therapy is a well-established treatment modality for patients with cancer. Ionizing radiation induces DNA damage, either directly (via charged particles) or indirectly (via free radical production) causing damage and dysfunction in both tumors and healthy cells [1–5]. However, radiation can also modulate intra- and intercellular signaling pathways related to a variety of cellular functions, e.g. proliferation (via e.g. p53-regulated cell cycle arrest), cell death (via e.g. apoptosis- or autophagy signaling pathways), invasiveness (via e.g. EGFR-mediated Akt signaling), angiogenesis (via e.g. VEGF-, IL6-, or HIF-1α signaling pathways), and immune response (via e.g. TGF-β signaling or cytokine activity) [2, 6–13]. Many of these functions are disrupted in tumor cells, resulting in different cellular radiosensitivity, which in turn may affect the outcome of the treatment.

Neuroendocrine tumors (NETs) of the small intestine are slow-growing malignant neoplasms that frequently metastasize. Patients often present with disseminated disease and symptoms of hormone overproduction [14]. In patients with NETs expressing somatostatin receptors (SSTRs), peptide receptor radionuclide therapy (PRRT) with radiolabeled somatostatin analog [^177^Lu]Lu-DOTATATE (^177^Lu-[DOTA^0^, Tyr^3^]-octreotate, trademarked Lutathera) is an option of adjuvant treatment after surgical tumor reduction. This approach has previously shown promising results, with significant induction of tumor regression, increased overall survival, and improved quality of life [15–17], and has been approved by the FDA and EMA for use in patients with gastroenteropancreatic NETs [18]. However, cure for patients with metastatic NETs is rarely achieved, and clinical results are modest in comparison with anti-tumor effects seen in NET animal models [19, 20]. As a consequence, there is a need for novel [^177^Lu]Lu-DOTATATE treatment optimization strategies, including rational combinations with other therapies [21–27]. To find suitable niches for increased anti-tumor effects of [^177^Lu]Lu-DOTATATE therapy in NETs, a better understanding of the radiobiological response in tumor tissue is needed [28, 29].

Most knowledge on therapeutic radiobiology is derived from studies on external beam radiation therapy, and little research has yet been performed on the radiobiology of PRRT [6]. However, the cellular mechanisms involved in radiation responses vary between different absorbed doses, dose rates, and types of radiation [6, 10, 30–35]. To determine the full extent of the radiobiological effect of [^177^Lu]Lu-DOTATATE therapy, it is important to include effects induced on the tumor microenvironment as well as systemic responses (e.g. effects on blood vessel structures, hormone balance and immune responses).

The human small intestine NET cell line, GOT1, was derived from a liver metastasis of a patient with metastatic small intestine NET [19]. One of the important characteristics of GOT1 is that it can be xenografted to nude mice. The GOT1 cells have retained important properties of NETs, including abundant expression of SSTR2 and SSTR5 [36, 37], and neuroendocrine markers chromogranin A, synaptophysin and serotonin [28]. Furthermore, [^177^Lu]Lu-DOTATATE has been shown to induce apoptosis in GOT1 xenografts, with a maximum apoptosis response at 1 and 3 d after injection [19].

The aim of this study was to characterize the proteomic response of GOT1 small intestine NET in nude mice following [^177^Lu]Lu-DOTATATE therapy, to identify possible venues for treatment optimization. A non-curative amount of [^177^Lu]Lu-DOTATATE was chosen to be able to obtain enough tumor tissue both early and during regrowth in order to reflect the clinical situation.

## Methods

### Animal experiments

GOT1 tumor xenografts were generated as previously described [38]. Briefly, 1 mm^3^ tissue samples of GOT1 tumor were transplanted s.c. in the neck of 4-week-old female BALB/c nude mice (CAnN.Cg-Foxn1nu/Crl, Charles River, Japan and Germany). Intraperitoneal injections of Domitor^®^ vet. (Orion Pharma Animal Health, Sweden), Ketaminol^®^ vet. (Intervet AB, Sweden), and Antisedan (Orion Pharma Animal Health, Sweden) were used for anesthesia during the transplantation procedure. Drinking water and autoclaved food were provided *ad libitum*.

Nineteen GOT1 tumor-bearing nude mice were included in the study. Fifteen animals were injected with 15 MBq [^177^Lu]Lu-DOTATATE (a non-curative treatment) into the tail vein and killed at 1 or 13 d (*n* = 3 and 12, respectively) after injection. For euthanasia, sodium pentobarbital (Sodium Pentobarbital vet., Apotek Produktion & Laboratorier AB, Sweden, 60 mg/mL) was administered intraperitoneally, resulting in deep anesthesia and loss of consciousness prior to death. Cardiac puncture was subsequently performed as a secondary physical method to ensure death and facilitate tissue collection. This method was chosen to provide rapid and humane euthanasia while preserving tissue integrity for downstream proteomic analyses. Time-points were chosen to represent the early tumor responses (1 d) and responses during tumor regrowth (13 d) after [^177^Lu]Lu-DOTATATE therapy [19, 28]. Four animals injected with saline solution were used as controls. During the study period, tumor volume measurements were performed at -1, 1, 3, 8, and 13 d after injection (*n* = 19, 14, 12, 12, and 12, respectively) using magnetic resonance imaging (MRI). Tumor tissue samples were excised and snap-frozen in liquid nitrogen and stored at -80 °C until proteomic analysis with liquid chromatography tandem-mass spectrometry (LC-MS/MS). Histological examination was performed on tumor tissue obtained within the same experimental study. The complete histopathological and multiparametric MRI analyses have been reported previously [39, 40]; the main histological findings relevant to interpretation of the present proteomic results are summarized below.

### Radiopharmaceutical

Preparation of [^177^Lu]Lu-DOTATATE (Lutathera™, IDB Holland, the Netherlands) was conducted according to the manufacturer’s instructions. Quality control was performed with instant thin layer chromatography (ITLCTM SG, PALL Corporation, USA) with a mobile phase consisting of 0.1 M sodium citrate (pH 5; VWR International AB, Sweden). The fraction of peptide-bound ^177^Lu was > 98% and the specific activity was ~ 26 MBq/µg octreotate. ^177^Lu activity in syringes was measured before and after injection using a well-type ionization chamber (CRC-15R; Capintec, USA).

### Dosimetry

The mean absorbed dose,$\:\:D\left({r}_{T},{T}_{D}\right)$, to the target tissue, $\:{r}_{T}$, was calculated according to

$$\:D\left({r}_{T},{T}_{D}\right)=\frac{\stackrel{\sim}{A}\left({r}_{S},\:{T}_{D}\right){\sum\:}_{i}{{E}_{i}Y}_{i}\phi\:\left({r}_{T}\leftarrow{r}_{S},\:{E}_{i},{T}_{D}\right)}{M\left({r}_{T},{T}_{D}\right)},$$

where $\:\stackrel{\sim}{A}\left({r}_{S},\:{T}_{D}\right)$ is the time-integrated activity in the source tissue, $\:{r}_{S}$, over the dose-integration period, *T*_*D*_ ($\:\stackrel{\sim}{A}({r}_{s},{T}_{D})={\int\:}_{0}^{{T}_{D}}A({r}_{s},t)\:dt$) [41]. *Ã* values were determined from previously published biodistribution data in GOT1 tumor-bearing mice receiving the same administered activity of 15 MBq [^177^Lu]Lu-DOTATATE [42]. In that study, the mean tumor activity concentrations were 1.6, 1.6, and 0.84%IA/g at 24, 72, and 168 h after administration, respectively, demonstrating prolonged retention of [^177^Lu]Lu-DOTATATE in GOT1 tumors. The corresponding mean tumor absorbed dose per administered activity, integrated to infinite time, was 0.27 Gy/MBq. These biodistribution data were used to determine the time-integrated tumor activity for the absorbed-dose calculations in the present study. The mean energy emitted per nuclear decay *i*, $\:{\sum\:}_{i}{\mathrm{E}}_{i}{\mathrm{Y}}_{i}$ (where E_*i*_ is the mean energy emitted per decay *i* and Y_*i*_ is the yield of emission *i*), was approximated to 147.9 keV/decay [43], including β-particles, Auger, and conversion electrons. $\:\phi\:\left({r}_{T}\leftarrow{r}_{S},\:{E}_{i},{T}_{D}\right)$=1 and $\:{r}_{T}={r}_{S}$ was assumed in all calculations. After dissection, the tumor tissue was weighed and the mass, $\:M\left({r}_{T},{T}_{D}\right)$, was determined.

### MRI examinations

Tumor volume determination was performed using a dedicated small animal 7T MRI system (Bruker BioSpin MRI GmbH, Germany; software: ParaVision 5.0) with a 72-mm transmit volume coil and a 4-channel array rat brain receiver coil (RAPID Biomedical GmbH, Germany) as previously described [44]. Briefly, a T2-weighted, 2-dimensional rapid acquisition with relaxation enhancement (RARE) sequence was used with respiration triggering and fat suppression. Voxel size was ~ 160*160*700 µm^3^ (contiguous slices). Images were processed using software developed in MATLAB (R2011b, The MathWorks, USA). All images were subjected to histogram equalization and a 3*3 median filter, and each tumor was manually delineated on all image slices to calculate the volume by multiplying the total number of voxels with the voxel volume.

### Sample preparation for LC-MS/MS

Tumor tissue samples were homogenized and solubilized in buffer solution (50 mM triethylammonium bicarbonate (TEAB, Fluka, Sigma Aldrich), 2% sodium dodecyl sulfate (SDS)) using a pistil in a 1.5mL tube. Protein concentration was estimated by the Pierce™ BCA Protein Assay (Thermo Scientific). Further, 30 µg of total protein extract per sample were chemically reduced by addition of DL-dithiothreitol (final concentration 100 mM) in order to break disulfide bonds between cysteines. These more accessible, not cross-linked, proteins were then subjected to filter-aided sample preparation modified from Wisniewski et al. [45]. For this, proteins were diluted four times with 8 M urea, applied onto Nanosep 30 K OMEGA filters (Pall Life Science), and washed with 8 M urea to remove SDS. Subsequently, 10 mM methyl methane thiosulfonate in digestion buffer (1% sodium deoxycholate, 50 mM TEAB) was used for alkylation. Proteins on filters were washed before and after alkylation with digestion buffer in order to remove reducing and alkylating reagents. The protein solutions were digested by trypsin (Pierce Trypsin Protease, MS Grade, Thermo Fisher Scientific) in a substrate: enzyme ratio of 100:1 (w: w) at 37 °C overnight, followed by a second incubation with trypsin, under the same conditions, for 4 h. Finally, the peptides were collected by centrifugation (13,800*g for 20 min). Protein quantification was performed using isobaric mass tagging. Each individual tumor sample was processed and labeled separately with one of the 10plex tandem mass tags (TMT) according to the manufacturer’s protocol (Thermo Scientific), thereby allowing relative protein quantification for each individual tumor. Following labeling, the individually labeled samples were combined into TMT 10plex sets for subsequent fractionation and LC-MS/MS analysis. In total, four TMT sets were used, with one common reference sample included in each set to enable comparison between TMT sets. The labeling reaction was stopped with 5% hydroxylamine, and peptides of each TMT 10plex set were pooled, concentrated, and acidified to pH 2 in order to precipitate SDC. High pH reversed-phase chromatography on an ÄKTA purifier (Thermo Scientific, column: Waters XBridge BEH C18 3.0 × 150 mm, 3.5 μm) was performed for fractionation of the complex peptide samples. For this, the pH was increased to basic conditions by the addition of ammonia, and peptides were eluted via a gradient from 10 mM ammonium formate pH 10.0 to 90% acetonitrile in 10mM ammonium formate. In total, 24 fractions were collected, which were pooled 1 + 13, 2 + 14, and so on until 12 + 24. The resulting 12 fractions of each TMT set were dried and resuspended in 20 µL 0.1% formic acid, 3% acetonitrile just before LC-MS/MS analysis.

### LC-MS/MS analysis

The 12 fractions of each TMT 10plex set were analyzed on an Orbitrap Fusion Tribrid mass spectrometer (MS) coupled to an Easy nanoLC1000 (Thermo Fisher Scientific). Injected peptides (1 µL) of each sample were separated on a self-packed analytical column (300 × 0.075 mm I.D.) packed with 3 μm Reprosil-Pur C18-AQ particles (Dr. Maisch, Germany) using an acetonitrile gradient (from 5% to 80% at 200 nL/min in 0.2% formic acid) over 60 min. Ions were injected into the mass spectrometer using a voltage of 1.8 kV and positive ion mode. MS scans were performed in an m/z range of 380–1200, at a resolution of 120,000, and MS/MS scans were accomplished in a data-dependent multinotch mode. Ions in each MS scan over threshold 10,000 were selected for fragmentation (MS2) by collision induced dissociation for identification at 30% and detection in the ion trap. Subsequent multinotch (simultaneous) isolation of the top 5 MS2 fragment ions was performed with a range of m/z 400–900. These fragments were selected for further fragmentation (MS3) by high energy collision dissociation at 55% and detection in the Orbitrap at a resolution of 60,000, an m/z range of 100–500. MS precursors were isolated in the quadrupole with a 1.6 m/z window. Furthermore, dynamic exclusion within 20 ppm during 30 s was used for m/z-values already selected for fragmentation.

### Database matching and relative TMT quantification

For protein identification and relative quantification, MS raw files of all fractions per TMT set were merged and processed by Proteome Discoverer version 1.4 (Thermo Fisher Scientific). The Mascot search engine (Matrix Science, Boston, MA, USA) using the Homo sapiens SwissProt database version May 2016 (Swiss Institute of Bioinformatics, Switzerland) was set on MS peptide tolerance of 5 ppm, and a fragment mass tolerance of 500 millimass units. Trypsin as cleaving enzyme was chosen allowing no miscleavages. Oxidation of methionines was selected as dynamic modification, and TMT labeling of lysines or peptide N-termini, as well as alkylation of cysteines was set as fixed modifications. A significance threshold of 1% false discovery rate was selected enabled by a search against a reverse database, and proteins identified were grouped by sharing the same sequences. For relative quantification, ratios of TMT reporter ion intensities (m/z 126–131) derived from the MS3 spectra were calculated. The intensity threshold for reporter ions was set on 2000; fragment ion tolerance of 3 mmu for the centroid peak with the smallest delta mass was selected. Only proteins with at least one unique peptide were considered for identification and quantification. Normalization of the quantification was performed on the protein median.

### Bioinformatic analysis

For significance testing of the treatment response, a t-test according to Welch was performed using R. For this, data were log transformed, treatment groups were selected, and significance values for protein regulation were calculated (*p* < 0.01, and a fold change < 1.5 (treated *versus* control)).

Functional annotation of proteins with significantly altered expression levels was performed using the DAVID bioinformatics resource tool [46]. Proteins were associated with biological processes using Gene Ontology terms (*p* < 0.05 using Fisher’s exact test).

Ingenuity Pathway Analysis (IPA; Ingenuity Systems/QIAGEN) was used to investigate biological functions, canonical signaling pathways, and potential upstream regulators associated with the proteomic response to [^177^Lu]Lu-DOTATATE. Proteins meeting the predefined criteria for differential expression (fold change ≥ 1.5 and *p* < 0.01) were analyzed separately for tumors collected at 1 and 13 d after treatment, together with their corresponding expression changes. Protein identifiers were mapped to molecules in the Ingenuity Knowledge Base and analyzed using the Core Analysis function. Enrichment of proteins within biological functions, canonical pathways, and upstream-regulator networks was evaluated using Fisher’s exact test, which estimates the probability that the observed overlap between proteins in the experimental dataset and molecules assigned to a given pathway or function in the Ingenuity Knowledge Base occurred by chance. Statistical significance was defined as *p* < 0.05. Where sufficient information on the direction of protein-expression changes and previously reported molecular relationships was available, IPA additionally calculated an activation z-score to predict activation or inhibition of biological functions, canonical pathways, and upstream regulators. A z-score > 2 was considered indicative of predicted activation and a z-score < − 2 of predicted inhibition [47].

### ELISA measurements

Tumor tissues were lysed in RIPA buffer containing protease and phosphatase inhibitors (ThermoFisher Scientific). To validate mass spectrometry results, levels of human ITPR2 (Inositol 1,4,5-trisphosphate receptor type 2) and PODXL (Podocalyxin) were measured by ELISA, following the instructions of the manufacturer (R&D systems and Invitrogen, respectively). Lysates were run in triplicates on a Victor3 1420 multilabel plate reader (Perkin Elmer, Waltham, MA, USA), at suitable dilutions determined in an optimization run, and standard curve was fitted using the four-parameter logistic regression model.

## Results

### [^177^Lu]Lu-DOTATATE induced a partial response in GOT1 tumors

**Fig. 1:**
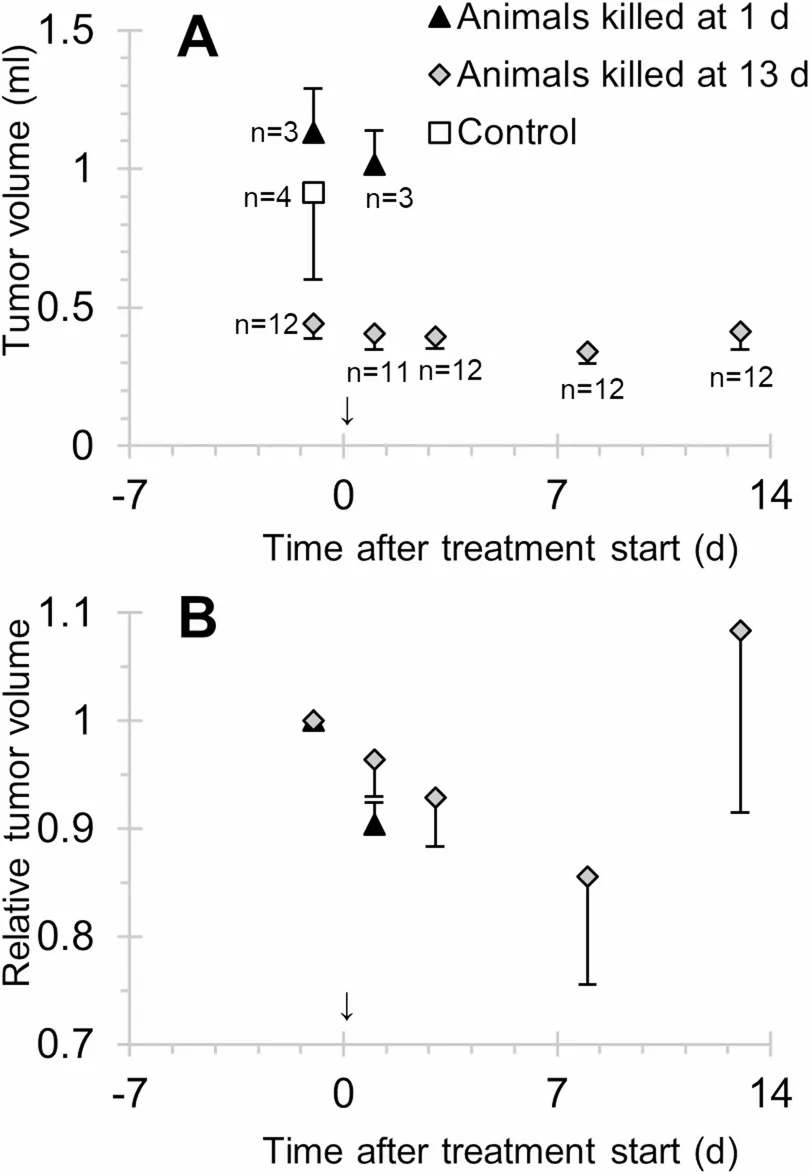
Anti-tumor effect of [^177^Lu]Lu-DOTATATE on GOT1 tumors in nude mice. The mean tumor volume (**A**) and relative tumor volume (**B**) versus time for mice killed at 1 d or 13 d after i.v. injection of 15 MBq [^177^Lu]Lu-DOTATATE. The number of animals measured is indicated by each data-point. Note the truncated y axis in Fig. 1B. Error bars indicate SEM. ↓ indicates the time for [^177^Lu]Lu-DOTATATE administration

Injection of 15 MBq [^177^Lu]Lu-DOTATATE resulted in mean absorbed doses to the tumors of 0.45 and 3.2 Gy calculated to 1 and 13 d, respectively, and 4.0 Gy when integrated to infinite time. These estimates were based on previously determined biodistribution data following administration of the same activity of 15 MBq [^177^Lu]Lu-DOTATATE in the GOT1 model, as described above [42]. Mean tumor volume relative to the day before injection was reduced in animals treated with [^177^Lu]Lu-DOTATATE (Fig. 1A-B). The minimum relative tumor volume (mean = 0.87, SEM = 0.12) was measured 8 d after injection in animals killed after 13 d. Microscopic examination of tumors at the end of experiment confirmed viable tumor cells in all experimental groups, and the microscopic appearance of tumors was characteristic for GOT1 tumors [39].

### Time-dependent proteomic responses in GOT1 tumors after [^177^Lu]Lu-DOTATATE therapy

**Fig. 2:**
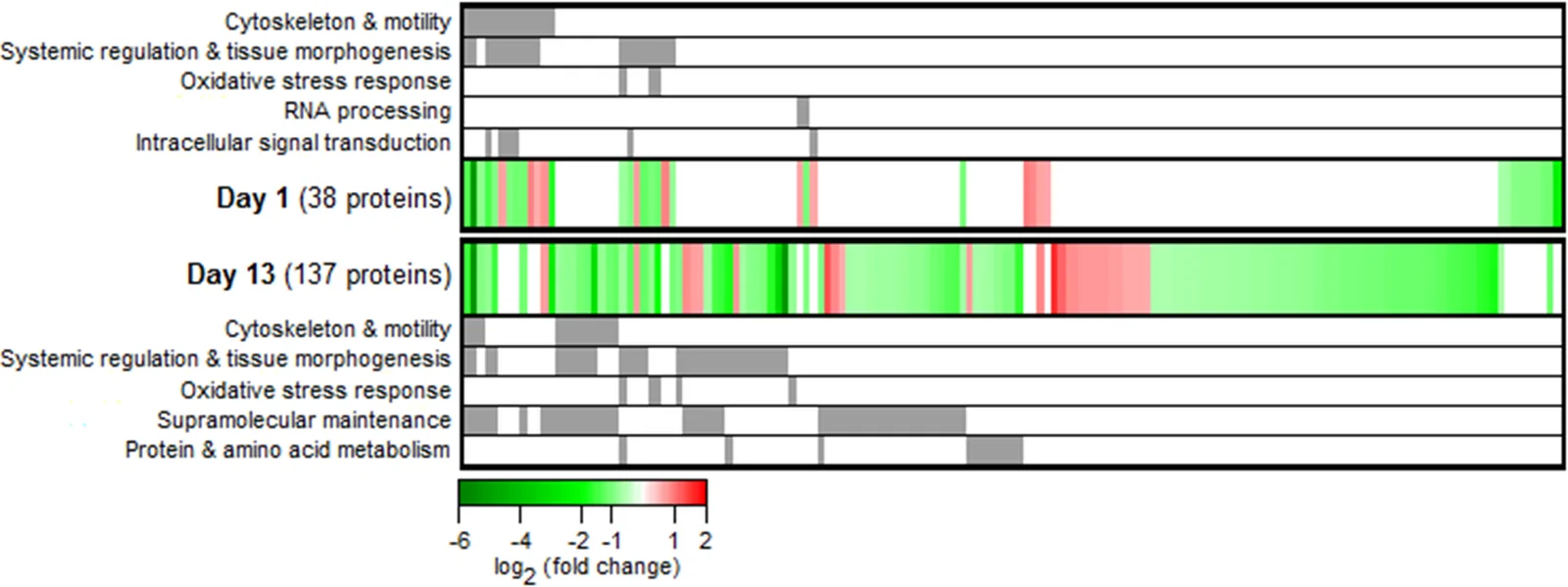
Distribution of proteins with altered protein levels in GOT1 tumors after [^177^Lu]Lu-DOTATATE administration. Alteration levels (log_2_(fold change)) of the 155 proteins significantly affected after i.v. injection of 15 MBq [^177^Lu]Lu-DOTATATE at 1 d or 13 d after injection, and annotation of enriched Gene Ontology terms. Red and green indicate elevated and decreased protein expression levels (treated *versus* control, fold change ≥ 1.5, *p* < 0.01), respectively. Gray indicates significant annotation of a protein to a specific Gene Ontology term (*p* < 0.05)

**Fig. 3:**
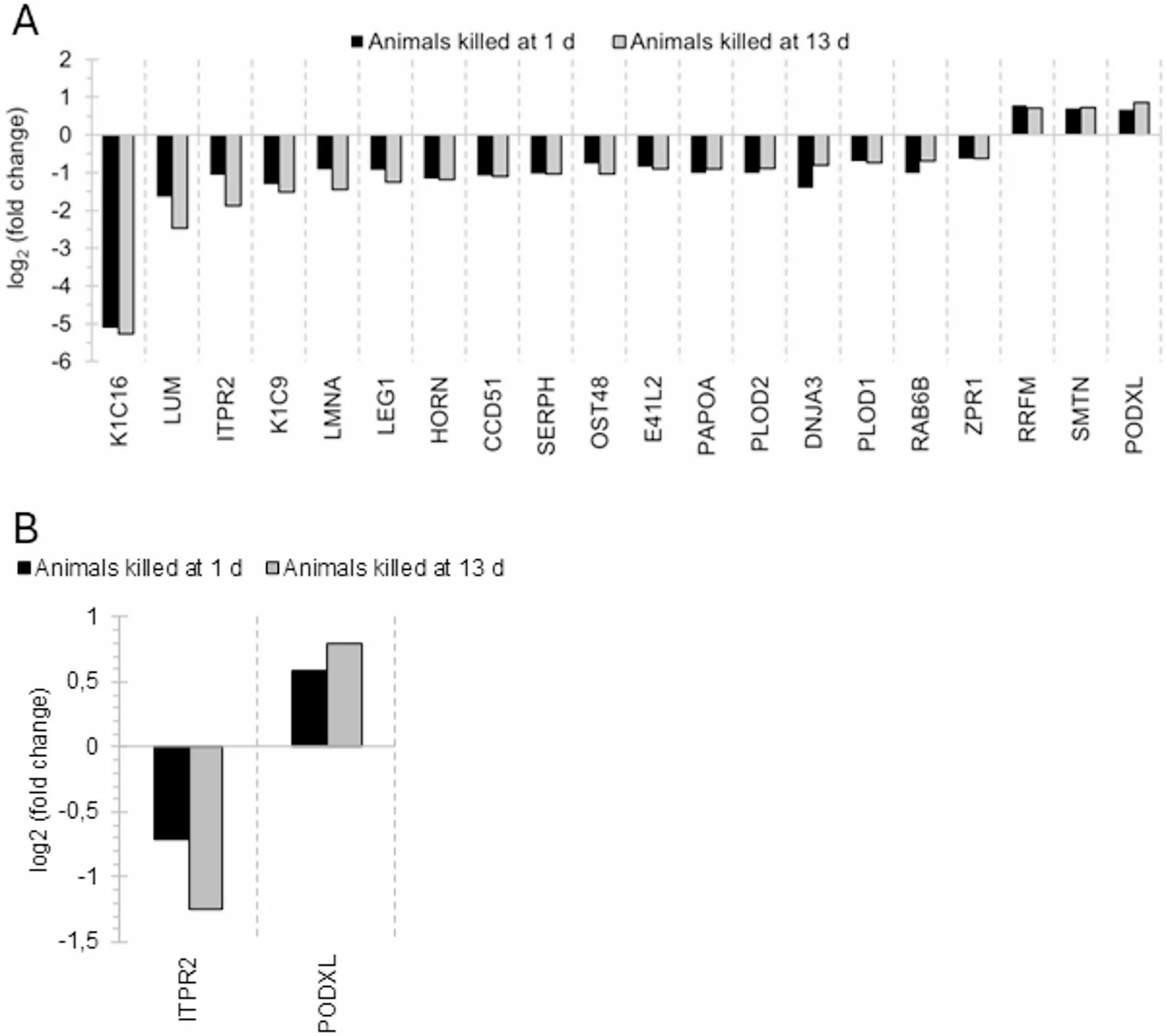
Proteins with altered expression in GOT1 tumors at both 1 d and 13 d after [^177^Lu]Lu-DOTATATE administration. Alteration levels (log_2_(fold change)) of the 20 proteins significantly affected after i.v. injection of 15 MBq [^177^Lu]Lu-DOTATATE at both 1 and 13 d after injection. Positive and negative numbers indicate elevated and decreased protein expression levels (treated *versus* control), respectively

A significant effect on protein expression levels was observed in the GOT1 tumors after administration of 15 MBq [^177^Lu]Lu-DOTATATE at both time-points studied. In total, 3861 different proteins were quantified in the LC-MS/MS analysis. Among these, 155 proteins had significantly altered expression levels at either 1 or 13 d after injection (fold change ≥ 1.5, *p* < 0.01, Fig. 2), and 20 proteins had significantly altered expression levels at both time-points (Fig. 3).

Annotation of the 155 proteins with altered expression to Gene Ontology terms revealed that C*ytoskeleton & motility*, *Systemic regulation & tissue morphogenesis*, and *Oxidative stress response* were affected at both time-points (*p* < 0.05, Fig. 2). Furthermore, *RNA processing* and *Intracellular signal transduction* were also affected early after injection, while *Supramolecular maintenance* and *Protein & amino acid metabolism* were affected during regrowth.

**Fig. 4:**
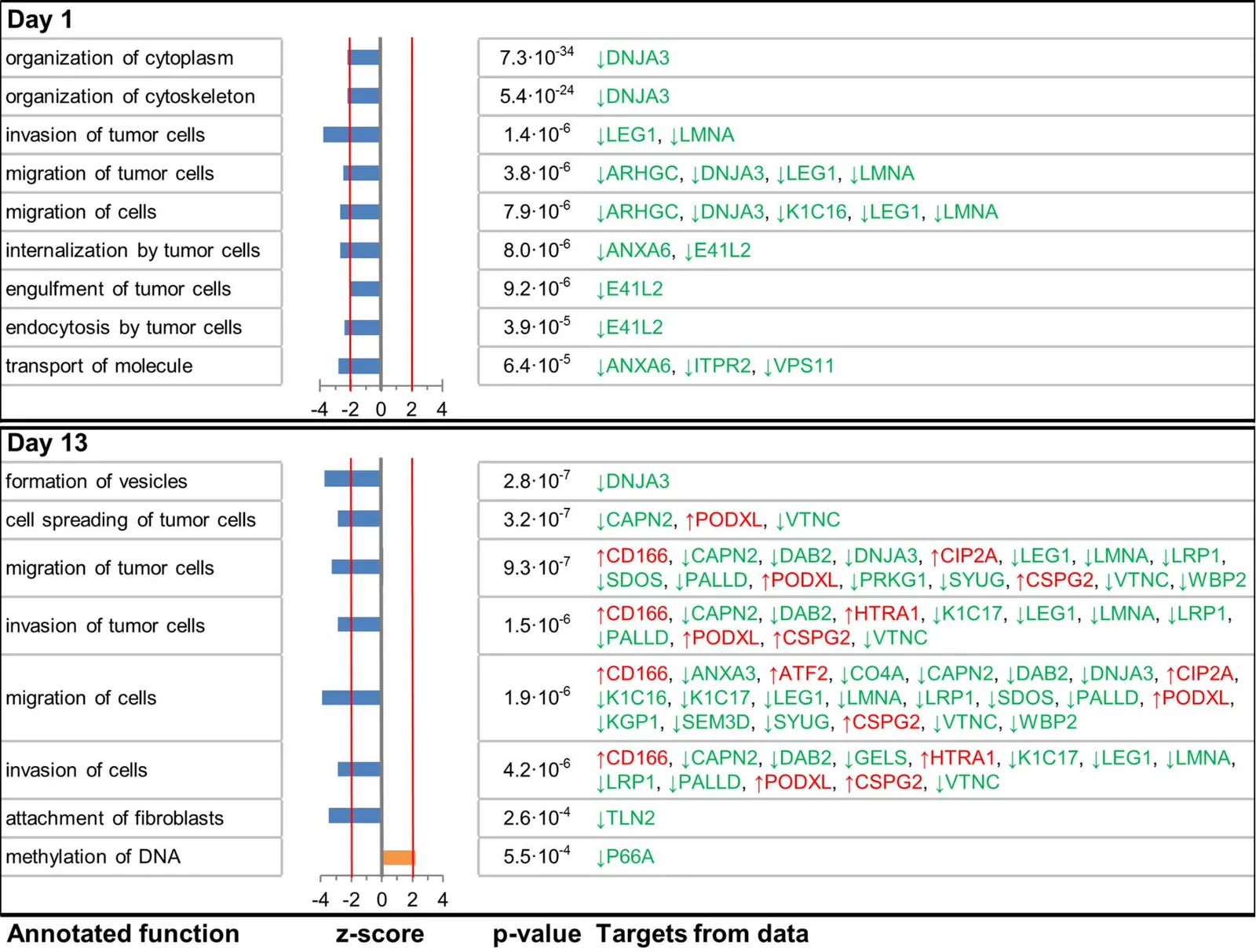
Predicted biological functions affected in GOT1 tumors after [^177^Lu]Lu-DOTATATE therapy. Significantly activated or inhibited biological functions identified with IPA (*p* < 0.05), ranked according to lowest p-value, for each time point. z-scores indicate activation state; z > 2 indicates activation (orange bars), z<-2 indicates inhibition (blue bars). Red↑ and green↓ indicate higher and lower tumor protein expression levels in treated samples compared with controls, respectively

Analysis of affected biological functions using IPA showed that a variety of functions related to tumor invasiveness and migration were significantly inhibited (z<-2, *p* < 0.05) both at day 1 (early tumor response), and at day 13 (tumor regrowth) after injection of [^177^Lu]Lu-DOTATATE. Proteins involved in this response included e.g. CD166, K1C17, LEG1, LMNA and VTNC (Fig. 4). Also inhibited early after injection were e.g. cytoskeleton organization, tumor internalization, and tumor endocytosis, while e.g. vesicle formation and methylation of DNA were inhibited and activated during regrowth, respectively.

### Differential effects on proteins related to cell morphology, cell cycle regulation and apoptosis in GOT1 tumors induced by [^177^Lu]Lu-DOTATATE therapy

**Fig. 5:**
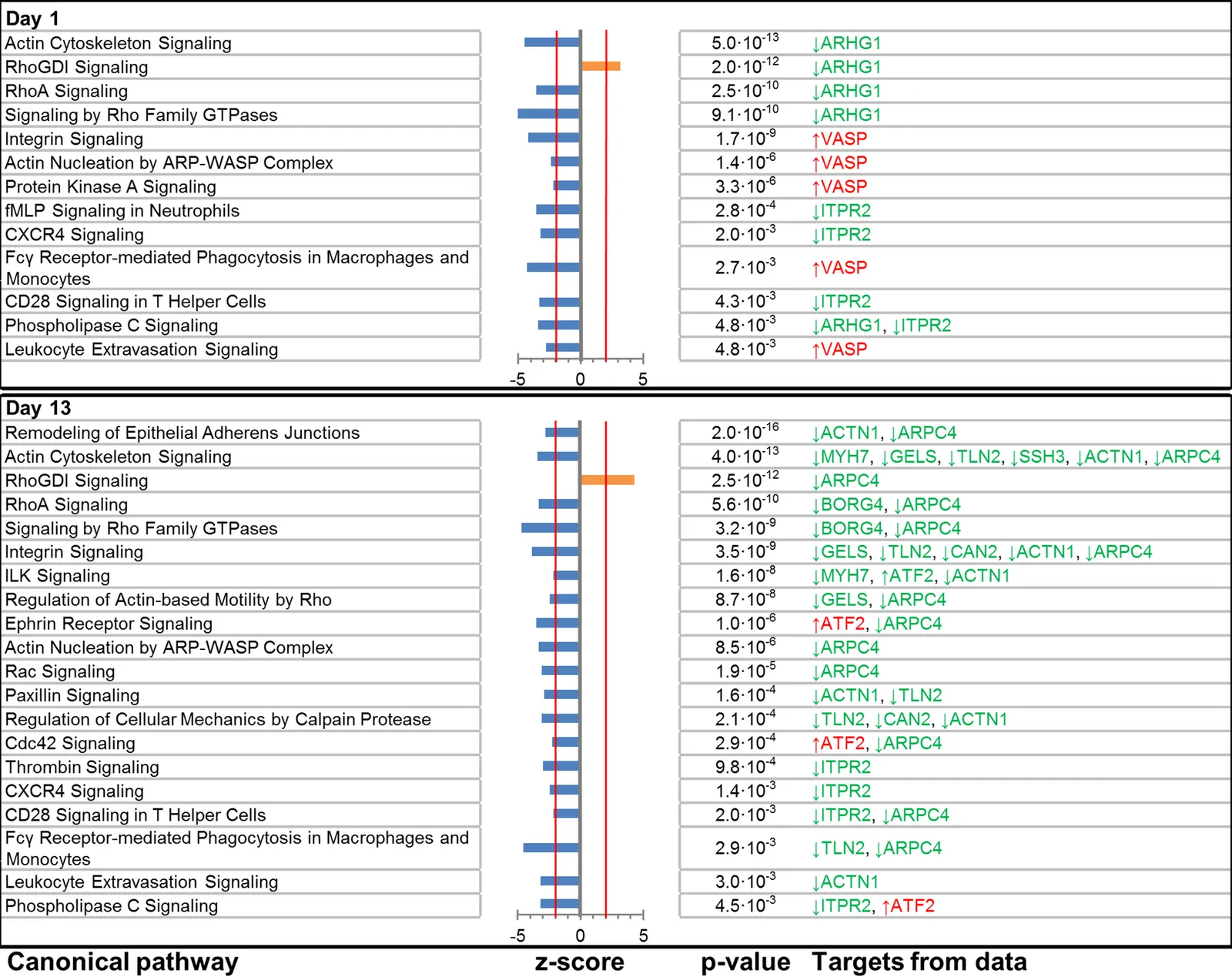
Predicted canonical pathways affected in GOT1 tumors after [^177^Lu]Lu-DOTATATE therapy. Significantly activated or inhibited canonical pathways identified with IPA (*p* < 0.05), ranked according to lowest p-value, for each time point. z-scores indicate activation state; z > 2 indicates activation (orange bars), z<-2 indicates inhibition (blue bars). Red↑ and green↓ indicate higher and lower tumor protein expression levels in treated samples compared with controls, respectively

Pathway analysis using IPA revealed a variety of significantly enriched canonical signaling pathways, with predicted activation states based on the direction of protein-expression change (*p* < 0.05, Fig. 5). Several pathways were related to Rho family of GTPases (a subfamily of the Ras superfamily, involved in cytoskeletal cell morphology) both early after injection of [^177^Lu]Lu-DOTATATE, and during regrowth, owing to the altered expression levels of e.g. ARHG1, ARPC4 and BORG4. Most of these Rho-related pathways were inhibited (z<-2, i.e. RhoA, Rac, Cdc42), while one (RhoGDI) was activated (z > 2) at both time-points. Several other pathways were inhibited at both time points, e.g. Actin cytoskeleton-, integrin-, CXCR4-, and phospholipase C signaling.

**Fig. 6:**
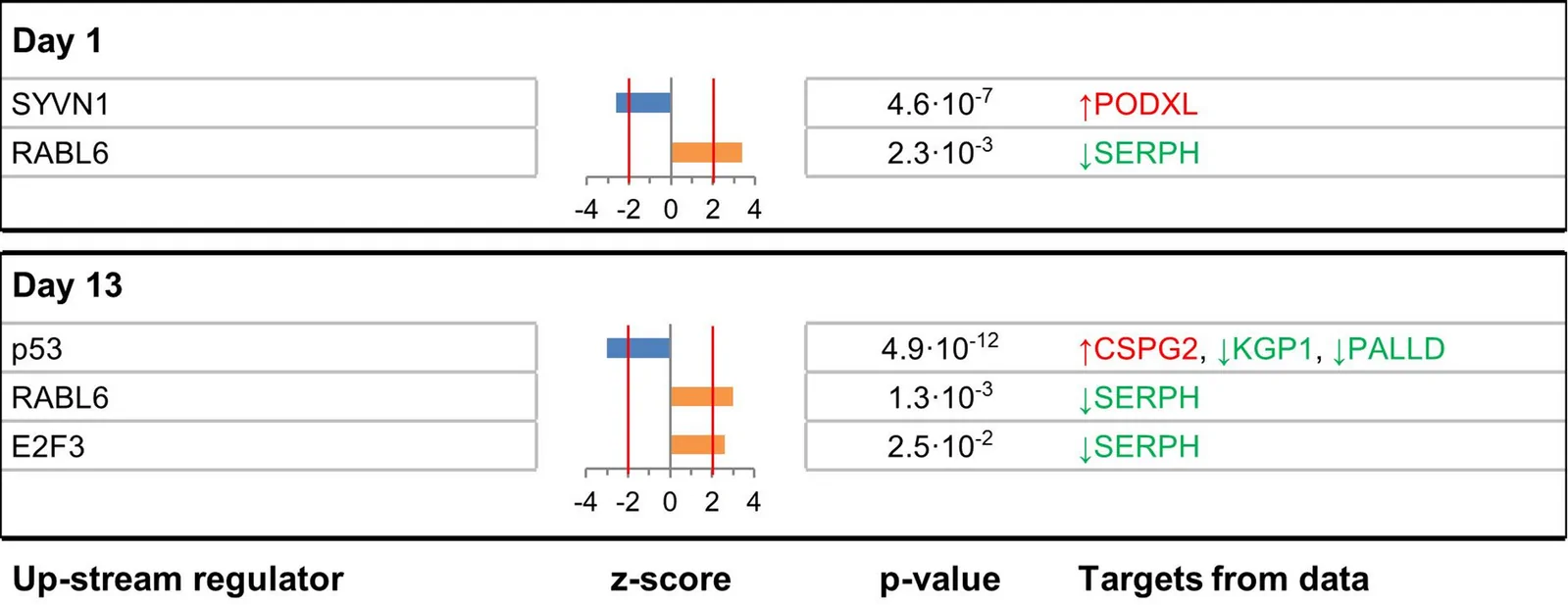
Predicted up-stream regulators in GOT1 tumors after [^177^Lu]Lu-DOTATATE therapy. Significantly activated or inhibited up-stream regulators identified with IPA (*p* < 0.05), ranked according to lowest p-value, for each time point. z-score indicates activation state; z > 2 indicates activation (orange bars), and z<-2 indicates inhibition (blue bars). Red↑ and green↓ indicate higher and lower tumor protein expression levels in treated samples compared with controls, respectively

Up-stream regulator analysis identified inhibition of SYVN1 (E3 ubiquitin-protein ligase synoviolin, involved in apoptosis inhibition) and activation of RABL6 (RAB member RAS oncogene family-like 6a, involved in cell cycle regulation) early after injection of [^177^Lu]Lu-DOTATATE (z-scores − 2.6 and 3.4, p-values 4.6·10^− 7^ and 2.3·10^− 3^, respectively, Fig. 6). During regrowth, p53 (regulator of e.g. DNA damage response) was identified as a significantly inhibited up-stream regulator (z-score − 3.0, p-value 4.9·10^− 12^). RABL6 and E2F3 (E2F transcription factor 3, functional in the control of cell-cycle progression from G1 to S phase) were also shown to be significant up-stream regulators activated during tumor regrowth (z-score 3.0 and 2.6, p-value 1.3·10^− 3^ and 2.5·10^− 2^, respectively).

### Protein expression validation

Protein quantification by ELISA performed for two of the proteins with altered levels at both 1 and 13 d after injection (ITPR2 and PODXL) validated the results from the LC-MS/MS analysis (Table 1).

**Table 1:** Alteration levels (log2(Fold change)) of the proteins ITPR2 and PODXL measured by LC-MS/MS and ELISA after i.v. injection of 15 MBq [^177^Lu]Lu-DOTATATE at both 1 and 13 d after injection. Positive and negative numbers indicate elevated and decreased protein expression levels (treated versus control), respectively

	log_2_(Fold change)	
	LC-MS/MS	ELISA
ITPR2		
1 d	-1,07	-1,01
13 d	-1,88	-1,87
PODXL		
1 d	0,66	0,68
13 d	0,86	0,85

## Discussion

This study presents proteomic responses of GOT1 human small intestine NET xenografts after 15 MBq [^177^Lu]Lu-DOTATATE therapy. On account of the differences in treatment efficacy between patient and animal studies [15, 16, 19, 20], mice were treated with a non-curative amount of ^177^Lu-octretotate to better reflect the clinical situation and enable analysis of both early tumor responses and responses during regrowth. The treatment resulted in an initial tumor volume reduction followed by tumor regrowth, in line with previously reported GOT1 tumor volume responses to [^177^Lu]Lu-DOTATATE [28, 40]. Therefore, we assume that the growth of the treated tumors was inhibited compared with growth rates in untreated GOT1 tumors (doubling time 14–16 d) [19, 36, 42].

To the best of our knowledge, this is the first study addressing the proteomic responses in tumor tissue after [^177^Lu]Lu-DOTATATE therapy in an in vivo setting. Twenty proteins showed altered expression levels both early after injection and during regrowth, compared with untreated controls. All of these alterations were in the same direction (17 downregulated and 3 upregulated), indicating a prolonged response over time. Gene Ontology term analysis revealed a significant enrichment of proteins related to previously identified cellular responses to radiation [7–9, 12], *e.g. Cytoskeleton & motility* (mainly constituted by proteins involved in actin-dependent motility, e.g. ARHG1, MYH7, GELS, and ACTN1), *Oxidative stress* (e.g. PDLIM1), *Systemic regulation & tissue morphogenesis* (constituted by proteins related to e.g. angiogenesis), *Supramolecular maintenance*, and *Protein and amino acid metabolism* (both terms constituted mainly by proteins related to ubiquitination and endoplasmic reticulum (ER) stress, e.g. NPL4, CDC23 and SERPH).

ER-stress is often caused by reactive oxygen species, which may be produced by radiation emitted by ^177^Lu (due to radiolysis of water), or downstream effects of the cellular damage caused by the irradiation [48]. ER-stress triggers the unfolded protein response (UPR), which initially acts to restore protein homeostasis by reducing general protein translation and targeting abnormal proteins for proteasomal degradation; however, prolonged or severe ER-stress may shift the UPR towards activation of apoptotic signaling [49, 50]. In line with these findings, SYVN1 (synoviolin/HRD1), an E3 ubiquitin ligase involved in ER-associated protein degradation and regulation of ER-stress responses and apoptosis [51], was predicted by IPA to be inhibited early after [^177^Lu]Lu-DOTATATE treatment. This may further support an effect of the treatment on ER protein homeostasis and apoptosis-related signaling. It has previously been reported that prolonged ER-stress causes an enhanced radiosensitivity in various tumors, due to a reduced capacity for double-strand break repair via Rad51 degradation through the ubiquitin-proteasome system [52]. The observed effects on ER-stress and the ubiquitin-proteasome system in the present study suggest that ER-stress-inducing treatments may be interesting for combination treatment with [^177^Lu]Lu-DOTATATE in this model.

Using IPA we identified a number of significantly affected biological functions which confirmed an inhibition of invasive and migratory signaling pathways, both as an early response and during tumor regrowth. This finding was unexpected, since previous studies have reported an enhanced metastatic potential in tumor cells following irradiation [7, 53]. However, the majority of these studies were performed with high energy photon irradiation, while there are studies showing that irradiation with carbon-ion beams can suppress invasive potential in tumors in vitro and in xenografts [7, 54]. In addition to differences constituted by different types of radiation, effects on tumor invasiveness may also vary depending on e.g. tumor type and rate of exposure. To our knowledge no studies of radiation-induced effects on invasiveness have been carried out in NETs, and no studies have investigated the effects of a prolonged exposure setting (as is employed in PRRT). There are reports of somatostatin-analog-induced suppression of cell invasion, although the effects of octreotate on invasive potential have not been studied [55]. Further studies are needed, in terms of amount of administered activity and choice of somatostatin analog, to determine the extent and mechanism of these effects.

Among the inhibited canonical pathways identified by IPA, several were correlated with metastatic formation (e.g. CXCR4-, Phospholipase C-, ILK-, integrin-, paxillin-, calpain,- and protein kinase A signaling) [53, 56–60]. RhoA- and Rac signaling have previously been implicated in invasiveness. The RhoA and Rac proteins function antagonistically and promote amoeboid or mesenchymal migration, respectively [61]. An inhibition of one of these in favor of the other would lead to a transition in motility mode, a capability which is important in metastasis formation. However, in the present investigation, inhibition of both RhoA and Rac was seen which may indicate a general suppression of migratory potential [62]. Several other inhibited pathways (i.e. actin cytoskeleton signaling, integrin signaling, actin nucleation by ARP-WASP complex, protein kinase A signaling, and remodeling of epithelial adherens junctions) have also been implicated in RhoA/Rac-dependent invasiveness [59, 61, 63]. The only significantly activated pathway (RhoGDI, z score 3.0 and 4.1 at 1 and 13 d after injection, respectively) has been shown to have inhibitory and degradation-protective effects on RhoA, indicating an inhibition of invasive potential [59]. Considering the target proteins that contribute to this predicted pathway responses, three were identified at 1 d after injection (ARGH1, VASP and ITPR2). The ARGH1 (Rho guanine nucleotide exchange factor 1) protein, involved in the activation of RhoA, and VASP (Vasodilator-stimulated phosphoprotein), is an actin-associated protein involved in cadherin binding and has been described as an effector protein to Rho GTPases [64]. The ITPR2 protein mediates calcium transport and has been implicated in response to hypoxia [65]. At 13 d after injection, several proteins (i.e. ACTN1, ARPC4, GELS, TLN2, SSH3, BORG4, all with lower expression levels after [^177^Lu]Lu-DOTATATE therapy) were identified as promoters of Rho GTPase-regulated motility and actin dynamics [61, 66, 67].

DNA damage and apoptosis are important components of the response to ionizing radiation [68, 69]. Previous studies in the GOT1 model demonstrated induction of apoptosis after [^177^Lu]Lu-DOTATATE treatment, with the strongest response observed at 1–3 d after administration [19]. In the present proteomic analysis, however, apoptosis and DNA damage repair were not among the significantly enriched biological functions or canonical pathways identified by IPA. This may partly reflect the selected sampling time points and the transient nature of these responses, particularly since the first time point represents an early response and the second tumor regrowth.

In order to identify alterations in key regulatory pathways after [^177^Lu]Lu-DOTATATE therapy, IPA upstream regulator analysis was performed. RABL6 was predicted to be activated at both 1 and 13 d after treatment based on the observed expression pattern of downstream proteins (Fig. 6). RABL6 has been reported to inhibit p53 activity in cells exposed to DNA damage and is overexpressed in several human cancers [70]. Consistent with this, p53 was predicted to be inhibited during tumor regrowth at 13 d. p53 is rarely mutated in NETs, but epigenetic and regulatory aberrations interfering with the p53 network activity have been described, which might inhibit p53 function [71–73]. Recovery of p53 function by targeting over-activated inhibitors may be a promising target to increase the anti-tumor effects of [^177^Lu]Lu-DOTATATE, in terms of enhancing p53-mediated cell cycle arrest and apoptosis. The activation of RABL6 is consistent with the inhibition of p53 during regrowth. These findings suggest that suppression of p53-related signaling may contribute to tumor recovery following [^177^Lu]Lu-DOTATATE-induced damage. However, since the predicted RABL6 and p53 activities were derived from pathway analysis and were not directly validated in the present study, further studies are required to establish their role in the radiation response.

It should be noted that the study was performed on a clinically relevant human tumor cell line, GOT1, transplanted on nude mice. By this in vivo study, many effects related to tumor microenvironment and systemic responses are included to better reflect the clinical situation. However, there are limitations that must be considered due to the immunodeficiency of the mice and the subcutaneous location of the tumors. Furthermore, the practical limitation of the animal model, with low tumor take and slowly growing tumors, results in few animals with suitable tumor sizes available at certain time-points. This situation causes limited number of tumors and differences in tumor size distribution between groups. Furthermore, only two time points were included in the proteomic analysis, selected to represent early treatment response and tumor regrowth. Additional intermediate time points would provide a more detailed characterization of the temporal proteomic response to [^177^Lu]Lu-DOTATATE. The differences in protein expression profiles and proteomic responses to [^177^Lu]Lu-DOTATATE treatment between tumors of different sizes are unknown, and since the treatment results in a change in tumor volume, the time-point after injection may also affect this possible difference. However, since 53% of the proteins with significantly altered expression at 1 d had an altered expression also at 13 d (with similar fold changes at the different time-points), the results from the proteomic analyses in these groups should be of importance to the understanding of the molecular responses to [^177^Lu]Lu-DOTATATE in the GOT1 model regardless of tumor size.

## Conclusions

In conclusion, the proteomic response of intravenous injection of 15 MBq [^177^Lu]Lu-DOTATATE revealed alterations of multiple biological functions and signaling pathways including suppression of invasive potential in GOT1 tumor xenografts. This may indicate that PRRT could have an advantage over conventional photon radiotherapy due to possible preventive effects on invasiveness and metastatic formation in irradiated malignant tumor cells. Predicted effects on p53-related cell cycle regulation, ER-stress, and oxidative stress suggest that combination therapy with [^177^Lu]Lu-DOTATATE and other anti-cancer agents such as p53-inhibitor antagonists or ER-stress-inducers may have favorable effects for treatment of NETs.

## Data Availability

The mass spectrometry proteomics data have been deposited to the ProteomeXchange Consortium via the PRIDE50 partner repository with the dataset identifier PXD074305.

## Abbreviations

ELISA: Enzyme-linked immunosorbent assay
ER: Endoplasmic reticulum
IPA: Ingenuity pathway analysis
LC-MS/MS: Liquid chromatography tandem-mass spectrometry
MRI: Magnetic resonance imaging
MS: Mass spectrometer
NET: Neuroendocrine tumor
PRRT: Peptide receptor radionuclide therapy
RARE: Rapid acquisition with relaxation enhancement
SSTR: Somatostatin receptor
TMT: Tandem mass tags
UPR: Unfolded protein response
